# Draft Genome Sequence of *Caloranaerobacter* sp. TR13, an Anaerobic Thermophilic Bacterium Isolated from a Deep-Sea Hydrothermal Vent

**DOI:** 10.1128/genomeA.01491-15

**Published:** 2015-12-17

**Authors:** Meixian Zhou, Yunbiao Xie, Binbin Dong, Qing Liu, Xiaoyao Chen

**Affiliations:** a Key Laboratory of Marine Genetic Resources, Third Institute of Oceanography, State Oceanic Administration, Xiamen, China; b Key Laboratory of Marine Genetic Resources of Fujian Province, Xiamen, China

## Abstract

Here, we report the draft 2,261,881-bp genome sequence of *Caloranaerobacter* sp. TR13, isolated from a deep-sea hydrothermal vent on the East Pacific Rise. The sequence will be helpful for understanding the genetic and metabolic features, as well as potential biotechnological application in the genus *Caloranaerobacter*.

## GENOME ANNOUNCEMENT

Thermophilic anaerobic microorganisms can thrive at temperatures over 50°C and do not require the use of O_2_ as a terminal electron acceptor for growth (1, 2). A growing number of anaerobic thermophilic bacteria have been isolated and studied from the deep-sea for their physiological properties and potential applications (3, 4). The anaerobic thermophilic bacterial genus *Caloranaerobacter* was first described by Wery et al. (5). It contains two type species: *Caloranaerobacter azorensis* MV1087 and *Caloranaerobacter ferrireducens* DY22619 (6). Recently, *Caloranaerobacter* spp. are reported to play an important role in biological hydrogen generation (7). *Caloranaerobacter* sp. TR13 (=MCCC 1A00790), an anaerobic, thermophilic, chemo-organotrophic bacterium, was isolated from a deep-sea hydrothermal vent sediment sample collected on the East Pacific Rise. It can grow at temperatures ranging from 37 to 75°C (optimum, 60°C), and at salinities of 0.5 to 7.0% (wt/vol) NaCl (optimum, 3.0% NaCl). Strain TR13 showed highest 16S rDNA sequence similarity with *C. ferrireducens* DY22619 (99.5%). To date, there is no reference *C. ferrireducens* genome sequence available publicly. Here, we report the draft genome sequence of strain TR13, the first released *C. ferrireducens* genome sequence. The sequence will be helpful for understanding the genetic and metabolic features, as well as potential biotechnological application in the genus *Caloranaerobacter*.

The genomic DNA of *Caloranaerobacter* sp. TR13 was extracted using a bacterial DNA kit (Omega) according to the manufacturer’s instructions. TR13 genome was sequenced by whole-genome shotgun sequencing using the Illumina MiSeq system at Shanghai Majorbio Bio-pharm Technology Co., Ltd. (Shanghai, China). A total of 346,433,714 reads were generated, representing approximately 153-fold coverage of the genome. These reads were assembled using SOAPdenovo v2.0 (8) and resulted in 52 contigs with an *N*_50_ of 259,980 bp. The longest contig size was 644,929 bp. The draft genome of strain TR13 was analyzed and annotated using the NCBI Prokaryotic Genome Annotation Pipeline (PGAP) (http://www.ncbi.nlm.nih.gov/genome/annotation_prok/).

The draft genome sequence of *Caloranaerobacter* sp. TR13 was 2,261,881 bp in total length, with a G+C content of 29.5%. The genome contains 2,170 predicted open reading frames (ORFs) and 2,126 predicted protein-coding sequences (CDSs). There were 21 tRNA genes, 3 rRNA genes (5S, 16S, and 23S), and 1 noncoding RNA (ncRNA) gene predicted from this assembly.

Strain TR13 could utilize yeast extract, tryptone, and peptone for its growth. Sequence analysis revealed that the TR13 genome encodes forty peptidases and twenty-six proteases including two subtilisin-like serine proteases. TR13 genome also encodes different types of amylases for polysaccharide hydrolysis. Compared to *C. azorensis* H53214 and *C. ferrireducens* DY22619, TR13 possesses more [FeFe] hydrogenase genes (seventeen) involved in biological hydrogen production (7). TR13 contains one superoxide reductase Sor, one rubredoxin, two rubrerythrin, two NADH oxidases, three disulfide oxidoreductases, and one thioredoxin reductase against oxygen stress (9). A heat shock protein GrpE and two chaperone DnaJ proteins in response to hyperosmotic and high temperature were also found in TR13 genome.

### Nucleotide sequence accession numbers.

This whole-genome shotgun project has been deposited at DDBJ/EMBL/GenBank under the accession number JXLL00000000. The version described in this paper is version JXLL01000000.
